# The Oncogenic and Tumor Suppressive Functions of the Long Noncoding RNA MALAT1: An Emerging Controversy

**DOI:** 10.3389/fgene.2020.00093

**Published:** 2020-02-27

**Authors:** Qingjuan Chen, Chenjing Zhu, Yingying Jin

**Affiliations:** ^1^ Department of Oncology, Yongchuan Hospital, Chongqing Medical University, Chongqing, China; ^2^ Department of Radiation Oncology, Jiangsu Cancer Hospital & Jiangsu Institute of Cancer Research & Affiliated Cancer Hospital of Nanjing Medical University, Nanjing, China; ^3^ Department of Oncology, The Second Affiliated Hospital of Xi’an Jiaotong University, Xi’an, China

**Keywords:** MALAT1, pre-messenger RNA splicing, gene expression, tumorigenesis, cell migration, invasion, metastasis

## Abstract

Long noncoding RNAs are recently emerging as critical factors of tumorigenesis. Originally regarded as a pre-messenger RNA (mRNA) splicing regulator, the long noncoding RNA MALAT1 has been demonstrated to regulate gene transcription by binding histone modification enzymes and transcription factors, and to regulate mRNA and protein expression post-transcriptionally by binding microRNAs (miRNAs) and acting as a sponge. Early studies consistently report that MALAT1 is up-regulated in human cancer tissues of various organ origins, particularly metastatic cancer tissues, that high levels of MALAT1 expression in cancer tissues are associated with poor patient prognosis, and that MALAT1 induces cancer cell proliferation, migration, and invasion *in vitro* and tumor metastasis in mice. By contrast, by analyzing multiple independent large datasets, MALAT1 have very recently been found to be down-regulated in human colorectal and breast cancer tissues, and low MALAT1 expression is associated with decreased patient survival. By binding to the transcription factor TEAD, MALAT1 suppresses metastasis gene expression, colorectal and breast cancer cell migration, invasion, and metastasis *in vitro* and in mice. MALAT1 has therefore been proposed to function as a tumor suppressor in colorectal and breast cancers. More comprehensive studies with multiple independent cohorts of human cancer tissues of various organ origins, *in vitro* and *in vivo* function, and mechanism studies with rescue experiments are required to confirm the oncogenic or tumor suppressive role of MALAT1 in other cancers.

## Introduction

### Long Noncoding RNAs

Noncoding RNAs account for approximately 98% of the transcription products of the human genome (Mattick and Gagen, 2001). Long noncoding RNA (lncRNAs) are RNAs > 200 nucleotides in length but do not possess protein-coding potential. While regarded as transcriptional junks for several decades, lncRNAs have become one of the most attractive subjects of medical research in the past several years (Quinn and Chang, 2016; Deveson et al., 2017). lncRNA are probably best classified according to their association with protein-coding genes and neighboring known DNA elements, messenger RNA (mRNA) resemblance, transcript length, sequence and structure conversation, subcellular structures, biochemical pathway or stability, as well as biological states and functions (St Laurent et al., 2015; Tang et al., 2017), although the 10 classification criteria are not mutually exclusive. LncRNAs are often expressed at lower levels compared to protein-coding RNAs in human tissues. However, with recent advances in next-generation RNA sequencing technology, a large number of new lncRNA transcripts have been uncovered (Quinn and Chang, 2016; Deveson et al., 2017).

LncRNAs suppress (Rinn et al., 2007; Kaneko et al., 2014; Kim et al., 2018) or activate (Wang et al., 2011; Gomez et al., 2013; Luo et al., 2019; Li et al., 2019) gene transcription by binding to chromatin remodeling protein complexes and by acting as transcriptional enhancers (Lai et al., 2013; Jiang et al., 2018). In addition, lncRNAs post-transcriptionally regulate mRNAs, such as pre-mRNA alternative splicing (Beltran et al., 2008; Andersen et al., 2019), mRNA stability (Yang et al., 2014; Liu et al., 2014), and translation into proteins (Yoon et al., 2012; Malakar et al., 2019; Liu et al., 2019).

### Long Noncoding RNAs as Oncogenes and Tumor Suppressors

Abnormal lncRNA expression plays important roles in tumor initiation and progression. A number of lncRNAs, such as HOTAIR (Gupta et al., 2010; Tsai et al., 2010; Kogo et al., 2011), ANRIL (Yap et al., 2010; Kotake et al., 2011), PANDA (Morris et al., 2008) (Hung et al., 2011), PCAT-1 (Prensner et al., 2011), NORAD (Lee et al., 2016), LncUSMycN (Liu et al., 2014), CCAT1 (Jiang et al., 2018), and LINC00673-v4 (Guan et al., 2019) are aberrantly over-expressed in human cancer tissues. On the other hand, a number of tumor suppressive lncRNAs, such as MEG3 (Zhou et al., 2007; Wang et al., 2012), LincRNA-p21 (Huarte et al., 2010; Luo et al., 2019), CASC15-S (Russell et al., 2015), and LINC00261 (Shahabi et al., 2019) are down-regulated in human cancer tissues. Aberrant expression of the oncogenic and tumor suppressive lncRNAs suppresses the transcription of tumor suppressor genes, enhances oncogene expression, suppresses the translation of tumor suppressor genes, and enhances oncogene translation, leading to cell proliferation, differentiation block, migration, invasion, metastasis, genomic instability, malignant transformation, tumor initiation, tumor progression, and resistance to chemo-radio-therapy. One of the most studied lncRNAs in cancer is metastasis-associated in lung adenocarcinoma transcript 1 (MALAT1), as MALAT1 is highly expressed in cancer tissues and easily detected and functionally analyzed.

### Introduction to MALAT1

Also known as nuclear enrichment autosomal transcript 2 (NEAT2), MALAT1 was initially identified through subtractive hybridization as one of the transcripts most significantly over-expressed in metastatic non-small cell lung cancer tissues (Ji et al., 2003). Human MALAT1 RNA consists of a single ~ 7 kb exon without an open reading frame for protein coding. Unlike the majority of lncRNAs, MALAT1 is very well evolutio*narily conserved* and very abundantly expressed in normal tissues (Hutchinson et al., 2007). While MALAT1 lncRNA undergoes epitranscriptomic modifications, including *N* ^6^-methyladenosine (Liu et al., 2017) and 5-methylcytosine (Amort et al., 2017), it is unknown whether these modifications affect its stability, expression level, or function. MALAT1 is mainly discovered in nuclear speckles (Tripathi et al., 2010) and has been reported to be involved in tumor metastasis (Ji et al., 2003). For example, N-Myc oncoprotein up-regulates the histone demethylase JMJD1A which in turn up-regulates MALAT1 in human neuroblastoma cells, and that MALAT1 induces tumor-driven endothelial cell migration, invasion, and angiogenesis as well as tumor cell migration, invasion, and metastasis (Tee et al., 2014; Tee et al., 2016).

## Mechanisms of Action of MALAT1

### MALAT1 Regulates Pre-Messenger RNA Splicing

The nuclear lncRNA MALAT1 can be recruited to nuclear speckles (Hutchinson et al., 2007), a site for pre-mRNA splicing factor storage and modification (Galganski et al., 2017). By binding pre-mRNA splicing factors, MALAT1 regulates their distribution in nuclear speckle domains and thereby modulates pre-mRNA splicing, mRNA expression, and cellular function (Tripathi et al., 2010). However, conclusions from the *in vitro* discoveries are challenged by the findings that no global changes in splicing factor levels or alternative pre-mRNA splicing can be observed in *MALAT1* knockout mice (Zhang et al., 2012; Eissmann et al., 2012; Nakagawa et al., 2012). Nevertheless, loss of *MALAT1* in mice leads to the dysregulation of a small number of mRNAs (Zhang et al., 2012) and the lncRNA NEAT1 (Nakagawa et al., 2012), which is an architectural component of nuclear bodies known as paraspeckles. In addition, loss of *MALAT1* in the mouse mammary tumor virus (MMTV)-PyMT mammary carcinoma mice results in alterations in the expression and splicing of genes important for cell differentiation and tumorigenesis (Arun et al., 2016). It can therefore be proposed that MALAT1 regulates pre-mRNA splicing in specific cells and tissues under particular conditions.

### MALAT1 Regulates Gene Transcription by Interacting With Histone Modification Enzymes and Transcription Factors

**Figure 1:**
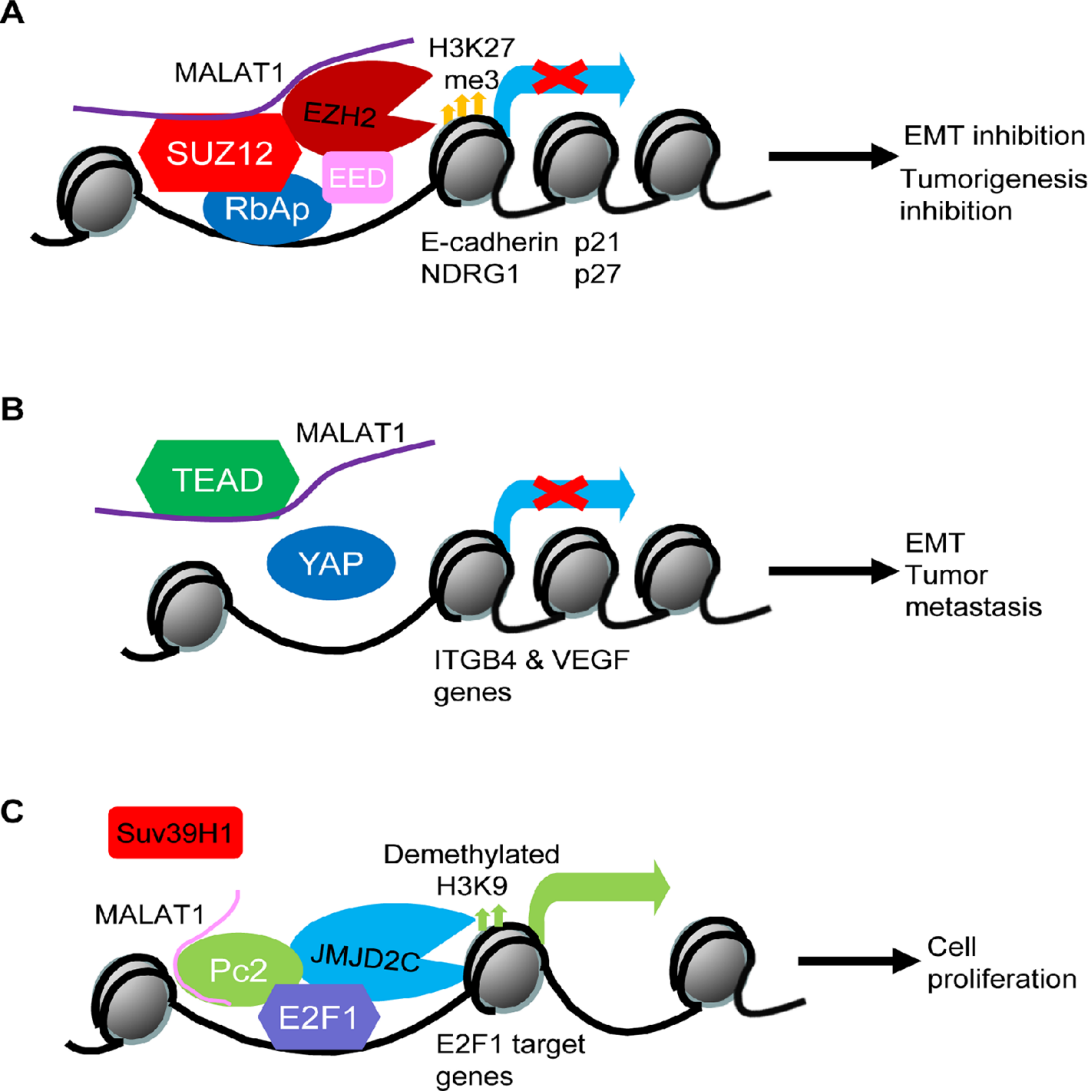
Models of MALAT1-mediated oncogenic and tumor suppressive effects through transcriptional regulation of oncogenes and tumor suppressors. **(A)** MALAT1 forms a RNA-protein complex with the Polycomb Repressive Complex 2 (PRC2) component proteins SUZ12 and EZH2 at the promoters of tumor suppressor genes such as E-cadherin, N-Myc downregulated gene-1 (NDRG1), p21, and p27, leading to histone H3K27 trimethylation, transcription repression, epithelial mesenchymal transition (EMT), and tumorigenesis. **(B)** MALAT1 binds and sequesters transcription factor TEAD proteins and thereby blocks TEAD proteins from associating with their co-activator YAP and the promoters of oncogenes such as integrin β4 *(*ITGB4*)* and vascular endothelial growth factor (VEGF), leading to oncogene transcriptional suppression and tumor suppressive effects. **(C)** After Pc2 protein is demethylated by the histone H3K9 demethylase JMJD2C, MALAT1 binds unmethylated Pc2 protein to promote E2F1 SUMOylation, transcription activation of E2F1 target genes, and cell proliferation.

When endogenous RNAs and their associated factors are purified from human cells, MALAT1 has been found to localize to hundreds of genomic sites, particularly of active genes, indicating that *MALAT1* might be involved in regulating gene transcription (West et al., 2014). Polycomb repressive complex 2 (PRC2) induces histone H3 lysine 27 (H3K27) tri-methylation and transcriptional repression of tumor suppressor genes (Laugesen et al., 2019). By forming a RNA-protein complex with EZH2 and SUZ12, two components of the PRC2 complex, MALAT1 increases histone H3K27 trimethylation at the promoters of tumor suppressor genes such as E-cadherin and N-Myc downregulated gene-1 (NDRG1) and suppresses their gene expression (Fan et al., 2014) (Hirata et al., 2015; Cheng et al., 2018). In addition, it has been shown that MALAT1 is required for PRC2 complex binding to gene promoters and consequent regulation of gene transcription, as MALAT1 knockdown dissociates EZH2 protein from the gene promoters of tumor suppressors, such as p21 and p27, and reverses EZH2-mediated gene silencing (Wang et al., 2016) (**Figure 1A**).

MALAT1 binds and sequesters the transcription factor TEAD proteins and thus blocks TEAD proteins from association with their co-activator YAP protein and target gene promoters, leading to transcriptional suppression of genes important for tumor metastasis, including integrin β4 (*ITGB4*) and vascular endothelial growth factor (VEGF) (Kim et al., 2018) (**Figure 1B**). MALAT1 has also been shown to form a RNA-protein complex with histone deacetylase HDAC9 and the bromodomain protein BRG1 in vascular smooth muscle cells, and the complex suppresses contractile protein gene expression (Lino Cardenas et al., 2018). In myoblast cells, MALAT1 recruits the histone lysine N-methyltransferase Suv39h1 to MyoD-binding loci at target gene promoters, causing histone H3K9 trimethylation and target gene silencing, and reduction in MALAT1 results in the destabilization of the Suv39h1/HP1β/HDAC1-repressive complex, allowing MyoD-mediated transcriptional activation (Chen et al., 2017).

Despite the above evidence for the role of MALAT1 in repressing gene transcription, MALAT1 has been found to display distinct gene body binding patterns at hundreds of active genomic sites in human cells (West et al., 2014), suggesting that MALAT1 may activate gene transcription. Demethylation and methylation of the Polycomb 2 (Pc2) protein regulates the relocation of cell proliferation genes between interchromatin granules (ICGs) and Polycomb bodies. Pc2 protein is methylated by Suv39h1 and demethylated by the histone H3K9 demethylase JMJD2C. Binding of MALAT1 to unmethylated Pc2 induces E2F1 protein SUMOylation, leading to the up-regulation of E2F1 target genes and cell proliferation (Yang et al., 2011) (**Figure 1C**).

### MALAT1 Regulates Messenger RNA and Protein Expression by Acting as a Competitive Endogenous RNA

Competitive endogenous RNAs (ceRNAs) have been proposed to sequester microRNAs so as to regulate the expression of mRNA transcripts containing common microRNA recognition elements (Karreth et al., 2011), and lncRNAs have often been assigned the role of ceRNAs. As an 8,708 bp lncRNA, *MALAT1* consist of a number of putative binding sites for microRNAs (miRNAs). To demonstrate the role of MALAT1 as ceRNAs, luciferase reporter assays are employed to confirm miRNA-binding to mRNAs and MALAT1 RNA, and functional experiments are employed to confirm the effect of MALAT1 on miRNA-regulated target mRNA and protein expression. While ceRNA model has been very attractive, there has always been skepticism, since recent transcriptome-wide studies on binding-sites suggest that physiological changes in most transcripts do not have significant effects on miRNA activity (Thomson and Dinger, 2016).

MALAT1 has been reported to bind a number of miRNAs and regulate miRNA function as a ceRNA. MALAT1 consists of sequence-specific miR-34a- and miR-22-binding sites, binds miR-34a and miR-22, and regulates miR-34a and miR-22 function, leading to up-regulation of c-Myc, Met, MMP14, and snail in melanoma cells (Luan et al., 2016; Li et al., 2019). In human colorectal cancer cells, MALAT1 expression is up-regulated by YAP1 (Sun et al., 2019), sponges miR-126-5p and miR-20b-5p, and thereby up-regulates the expression of tumor cell stemness proteins such as Oct4 and Nanog and metastasis-associated molecules such as VEGF, *SNAI2,* and TWIST (Sun et al., 2019; Tang et al., 2019). In ovarian cancer cells, MALAT1 sponges miR-211, thus up-regulating PHF19 expression and facilitating ovarian cancer progression (Tao et al., 2018) (**Table 1**).

**Table 1:** Oncogenic and tumor suppressive functions of MALAT1.

Binding protein/miRNA	Functions	References
	**Oncogenic**	
EZH2	Suppresses E-cadherin and increases β-catenin and c-Myc expression, induces renal cell carcinoma cell survival, proliferation, and invasion	(Hirata et al., 2015)
EZH2	Decreases p21 and p27 expression, leading to mantle cell lymphoma cell survival, proliferation and colony formation	(Wang et al., 2016)
SUZ12	Decreases E-cadherin and increases N-cadherin and fibronectin, leading to EMT, bladder cancer cell migration, invasion, and metastasis	(Fan et al., 2014)
miR-34a and miR-22	Up-regulates c-Myc, Met, MMP14, and snail expression, and promotes melanoma cell proliferation, migration, and invasion *in vitro* and tumor progression in mice	(Luan et al., 2016; Li et al., 2019)
miR-126-5p and miR-20b-5p	Up-regulates Oct4, Nanog, VEGF, SNAI2, and TWIST, and enhances colorectal cancer stem cell self-renewal, colorectal cancer cell proliferation, migration, metastasis *in vitro*, and tumorigenicity in mice	(Sun et al., 2019; Tang et al., 2019)
miR-211	Up-regulates PHF19 and RBFOX2 expression and alternative processing of the pro-apoptotic KIF1B, and induces ovarian cancer cell proliferation, invasion, anchorage-independent growth, and anoikis	(Tao et al., 2018)
miR-200c-3p and miR-217	Up-regulates oncogenic ZEB1 and KRAS expression, and induces pancreatic cancer cell migration and invasion	(Liu et al., 2017; Zhuo et al., 2018)
miR-206	Up-regulates ANXA2 and KRAS, and promotes gallbladder cancer cell proliferation and invasion *in vitro* and tumor progression in mice	(Wang et al., 2016)
miR-1, miR-124, and miR-448	Reduces CDC42 and up-regulates CDK4 expression, and induces breast cancer cell cycle progression, cell migration, and invasion	(Chou et al., 2016; Feng et al., 2016; Bamodu et al., 2016)
miR145-5p	Decreases miR145-5p, increases NEDD9 protein, and induces non-small cell lung cancer cell migration and invasion	(Yu et al., 2019)
miR-200c	Promotes TGFβ-induced endometrioid endometrial carcinoma cell EMT, migration, and invasion	(Li et al., 2016)
miR-23b-3p	Up-regulates ATG12 autophagy protein, and induces gastric cancer cell autophagy and chemoresistance	(Yiren et al., 2017)
miR-216b	Promotes autophagy and hepatocellular carcinoma cell resistance to chemotherapy agents 5-fluorouracil, Adriamycin, and mitomycin C	(Yuan et al., 2016)
EZH2	Suppresses E-cadherin expression and promotes oxaliplatin-induced EMT and colorectal cancer cell resistance to oxaliplatin	(Li et al., 2017)
	**Tumor suppressive**	
PTEN	Suppresses colorectal and breast cancer cell migration and invasion by down-regulating the pro-metastatic Epithelial Cell Adhesion Molecule (EpCAM) and ITGB4	(Kwok et al., 2018)
TEAD	Preventing TEAD association with YAP, down-regulates YAP and TEAD target genes *ITGB4* and VEGF expression, and suppresses breast cancer cell migration, invasion, and metastasis	(Kim et al., 2018)

MALAT1 RNA is degraded by miR-200c-3p and miR-217 in pancreatic cancer cells. Conversely, MALAT1 functions as a ceRNA to suppress miR-200c-3p and miR-217 function and inhibit the translocation of miR-217 from the nucleus to the cytoplasm, leading to ZEB1 and KRAS upregulation (Liu et al., 2017; Zhuo et al., 2018). By sponging miR-206, MALAT1 up-regulates ANXA2 and KRAS expression in gallbladder cancer cells (Wang et al., 2016), and reduces CDC42 and up-regulates CDK4 expression in breast cancer cells (Chou et al., 2016; Feng et al., 2016; Bamodu et al., 2016) (**Table 1**).

In non-small cell lung cancer cells, MALAT1 binds miR145-5p and decreases its expression, leading to increased NEDD9 protein expression, since miR145-5p targets the 3'-untranslated region (UTR) of NEDD9 mRNA (Yu et al., 2019). In human endometrioid endometrial carcinoma cells, MALAT1 binds and sponges miR-200c, represses miR-200c expression and function, and is required for transforming growth factor β (TGFβ) function (Li et al., 2016). In addition, MALAT1 binds and sponges miR-216b in fluorouracil-resistant hepatocellular carcinoma cells (Yuan et al., 2016), and acts as a ceRNA for miR-23b-3p in chemotherapy-resistant gastric cancer cells (Yiren et al., 2017) (**Table 1**).

While the above MALAT1-miRNA interaction has been supposed to promote cancer, MALAT1 has been reported to exert tumor suppressive effects by sponging miRNAs miR‐17, 20a, and 106b and thereby decreasing the expression of epithelial cell adhesion molecule (EpCAM) and ITGB4 in colon and breast cancer cells (Kwok et al., 2018) (**Table 1**).

Despite the above wealth of literature, MALAT1 is enriched in the nucleus, and miRNAs align the RNA-induced silencing complex to target mRNAs and bind at complementary seed sequences of target mRNAs in the cytoplasm (Bartel, 2009). As such, the role of MALAT1 as a ceRNA needs to be interpreted with caution.

## The Controversy: Oncogenic and Tumor Suppressive Functions of MALAT1

### Evidence That MALAT1 Induces Cancer Cell Proliferation, Survival, Migration, Invasion, and Metastasis

MALAT1 is over-expressed in human mantle cell lymphoma and renal cell carcinoma tissues, compared with normal counterparts, and high levels of MALAT1 are associated with advanced disease stage and reduced patient survival (Hirata et al., 2015; Wang et al., 2016). MALAT1 is transcriptionally activated by c-Fos in renal cell carcinoma cells (Hirata et al., 2015). By interacting with the PRC2 component protein EZH2 and facilitating histone H3K27 trimethylation, MALAT1 suppresses p21, p27, and E-cadherin expression and thereby increases β-catenin and c-Myc expression, leading to mantle cell lymphoma and renal cell carcinoma cell survival, proliferation, and invasion (Hirata et al., 2015; Wang et al., 2016). Similarly, in bladder cancer cells, MALAT1 gene expression is activated by TGFβ (Fan et al., 2014). By association with the other PRC2 component protein SUZ12, MALAT1 decreases E-cadherin expression and increases N-cadherin and fibronectin expression, leading to epithelial mesenchymal transition (EMT), bladder cancer cell migration, and invasion *in vitro* and tumor metastasis in animal models (Fan et al., 2014). In human bladder cancer tissues, MALAT1 gene expression negatively correlates with E-cadherin expression and a high level of MALAT1 significantly correlates with poor patient survival (Fan et al., 2014) (**Table 1**).

MALAT1 has also been shown to promote cancer through functioning as a ceRNA. MALAT1 is over-expressed in human melanoma compared to adjacent normal tissues, and MALAT1 expression negatively correlates with miR-34a expression in melanoma tissues (Luan et al., 2016; Li et al., 2019). By binding miR-34a and miR-22 and regulating miR-34a and miR-22 function, MALAT1 up-regulates c-Myc, Met, MMP14, and snail expression, promotes melanoma cell proliferation, invasion, and migration *in vitro*, and is required for melanoma progression in mice (Luan et al., 2016; Li et al., 2019). In human colorectal cancer cells and tissues, MALAT1 expression positively correlates with YAP1, negatively correlates with miR-126-5p and miR-20b-5p expression, and predicts poor patient prognosis (Sun et al., 2019; Tang et al., 2019). By sponging miR-126-5p and miR-20b-5p, MALAT1 up-regulates the expression of tumor cell stemness proteins such as Oct4 and Nanog and metastasis-associated molecules such as VEGF, SNAI2, and TWIST, and thereby enhances colorectal cancer stem cell self-renewal, colorectal cancer cell proliferation, migration, metastasis *in vitro*, and tumorigenicity in mice (Sun et al., 2019; Tang et al., 2019). In human ovarian cancer tissues, high MALAT1 expression is associated with advanced disease stage, recurrence, and reduced survival (Gordon et al., 2019). By sponging miR-211, MALAT1 up-regulates PHF19 expression and induces RBFOX2 expression and alternative processing of the tumor suppressor gene KIF1B, leading to ovarian cancer cell proliferation, invasion, increased anoikis, and anchorage-independent growth (Tao et al., 2018; Gordon et al., 2019) (**Table 1**).

In human pancreatic ductal adenocarcinoma tissues, MALAT1 expression positively correlates with the oncogenic ZEB1 and negatively correlates with miR-200c-3p (Zhuo et al., 2018), and MALAT1 is expressed at higher levels in metastatic compared with localized tumors (Liu et al., 2017). Multivariate analysis reveals that high levels of MALAT1 expression in human pancreatic ductal adenocarcinoma tissues independently predict poor patient survival (Zhuo et al., 2018). While miR-200c-3p and miR-217 induce MALAT1 RNA degradation, MALAT1 suppresses miR-200c-3p and miR-217 function and miR-217 translocation from the nucleus to the cytoplasm, up-regulates ZEB1 and KRAS expression, and induces pancreatic cancer cell migration and invasion (Liu et al., 2017; Zhuo et al., 2018). It is worth noting that the effects of MALAT1 on pancreatic cancer cell proliferation is controversial, as the two studies report growth promoting (Liu et al., 2017) and no effect (Zhuo et al., 2018) respectively. MALAT1 is overexpressed in human gallbladder cancer tissues, and high levels of MALAT1 expression correlate with larger tumor size, lymphatic metastasis, and poorer overall survival. By functioning as a ceRNA for miR-206, MALAT1 up-regulates the expression of the oncogenic ANXA2 and KRAS, and promotes gallbladder cancer cell proliferation and invasion *in vitro* and tumor progression in mice (Wang et al., 2016).

In human breast cancer tissues, MALAT1 has been reported to be aberrantly up-regulated and high levels of MALAT1 expression correlate with poor patient prognosis (Chou et al., 2016; Feng et al., 2016; Wang et al., 2018). MALAT1 binds miR-1, miR-124, and miR-448, and acts as a ceRNA to reduce CDC42 and up-regulate CDK4 expression, leading to breast cancer cell cycle progression, cell migration, and invasion (Chou et al., 2016; Feng et al., 2016; Bamodu et al., 2016). Genetic loss or treatment with antisense oligonucleotides targeting MALAT1 results in alterations in the expression and splicing patterns of genes important for breast cancer cell differentiation, migration and oncogenesis; and results in tumor cell differentiation, slower tumor growth, and less metastasis in the *MMTV-PyMT* model of mouse mammary carcinoma (Arun et al., 2016). In lung cancer, MALAT1 actively regulates the expression of a set of metastasis-associated genes, MALAT1-deficient cells show decreased migration and form fewer tumor nodules in mice, and antisense oligonucleotides targeting MALAT1 prevent metastasis formation after tumor cell implantation in mice (Gutschner et al., 2013). In addition, in non-small cell lung cancer cells, estrogen receptor β increases MALAT1 gene transcription by binding the estrogen response elements at the MALAT1 gene promoter. MALAT1 binds miR145-5p and decreases its expression, resulting in increased NEDD9 expression, as miR145-5p targets NEDD9 mRNA 3'-UTR, and cancer cell migration and invasion (Yu et al., 2019) (**Table 1**).

Data on the role of MALAT1 in endometrioid endometrial carcinoma is inconsistent. In human endometrioid endometrial carcinoma tissues, miR-200c levels are higher and MALAT1 levels are lower in tumor than non-tumor tissues. MALAT1 binds and sponges miR-200c, and is required for TGFβ-induced endometrioid endometrial carcinoma cell EMT, migration, invasion (Li et al., 2016) (**Table 1**).

### MALAT1 in Drug Resistance

MALAT1 has been reported to play a role in chemotherapy-resistance. Compared with parental counterparts, chemotherapy-resistant gastric cancer cells show higher levels of MALAT1 and autophagy. By acting as a ceRNA for miR-23b-3p, MALAT1 suppresses miR-23b-3p-mediated ATG12 autophagy protein reduction, and induces gastric cancer cell autophagy and chemoresistance (Yiren et al., 2017). Silencing of MALAT1 suppresses chemotherapy-induced autophagy and sensitizes gastric cancer cells to chemotherapeutics (Yiren et al., 2017). Similarly, MALAT1 expression is over two folds higher in 5-fluorouracil-resistant than parental hepatocellular carcinoma cells. By binding and down-regulating miR-216b, MALAT1 promotes autophagy and renders hepatocellular carcinoma cell resistance to chemotherapy agents 5-fluorouracil, Adriamycin, and mitomycin C (Yuan et al., 2016) (**Table 1**).

In colorectal cancer tissues from patients receiving oxaliplatin-based chemotherapy, high levels of MALAT1 correlate with resistance to oxaliplatin treatment and poor patient survival (Li et al., 2017). MALAT1 binds to EZH2 and suppresses E-cadherin expression and promotes oxaliplatin-induced EMT and colorectal cancer cell resistance to oxaliplatin (Li et al., 2017) (**Table 1**).

### MALAT1 as a Tumor Suppressor

In direct contrast to the above observations, two recent comprehensive studies reveal that MALAT1 exerts tumor suppressive effects against colorectal and breast cancers. By analyzing seven independent human colorectal cancer tissue gene expression-patient prognosis datasets, Kwok *et al.* have found that MALAT1 gene expression positively correlates with the expression of the tumor suppressor PTEN, and that decreased expression levels of MALAT1 and PTEN are significantly associated with increased mortality in colorectal cancer patients (Kwok et al., 2018). Consistent with these data, in human breast cancer tissues, MALAT1 expression positively correlates with PTEN expression, and high levels of MALAT1 are associated with increased patient survival (Kwok et al., 2018). Mechanistically, PTEN up-regulates MALAT1 expression by binding and sequestering miR-17, miR-20a and miR-106b, and MALAT1 suppresses colorectal and breast cancer cell migration and invasion by reducing the pro-metastatic Epithelial Cell Adhesion Molecule (EpCAM) and ITGB4 (Kwok et al., 2018).

Analysis of RNA sequencing data from The Cancer Genome Atlas (TCGA) datasets and an Oncomine data-mining platform reveal that MALAT1 is expressed at a significantly lower level in human breast cancer than normal mammary tissues, at a significantly lower level in higher-grade than lower-grade breast cancer tissues, and at a significantly lower level in metastatic than primary breast cancer tissues (Kim et al., 2018). Kaplan-Meier (KM) survival analysis shows that a lower level of MALAT1 in breast cancer tissues correlates with shorter distant metastasis-free survival (Kim et al., 2018). *In vitro* studies show that MALAT1 forms a direct RNA-protein complex with the pro-metastatic transcription factor TEAD, prevents TEAD from associating with its co-activator YAP, suppresses the binding of YAP and TEAD to the promoters of their target genes, such as *ITGB4* and VEGF, decreases YAP-TEAD target gene expression, and suppresses breast cancer cell migration and invasion (Kim et al., 2018). Importantly, compared with control PyMT mouse mammary tumors, MALAT1-deficient PyMT mouse mammary tumors exhibit increased YAP-TEAD target gene expression and tumor metastasis, which can be reversed by genetic MALAT1 add-back (Kim et al., 2018).

## Conclusions

Due to its localization to nuclear speckles and binding to pre-mRNA splicing factors, MALAT1 has initially been proposed to play an important role in pre-mRNA splicing and consequently mRNA expression. However, studies with mouse models suggest that MALAT1 modulates pre-mRNA splicing only in specific cells and tissues under particular conditions. Through forming RNA-protein complexes with histone modification enzymes, such as the PRC2 components EZH2 and SUZ12, and transcription factors, such as Pc2 and TEAD, MALAT1 suppresses and enhances the transcription of genes important for cancer cell proliferation, migration, invasion, and metastasis. Additionally, MALAT1 binds a number of miRNAs and functions as a ceRNA, and thereby regulates the expression of miRNA target mRNAs and proteins, cancer cell proliferation, migration, invasion, and metastasis.

Early studies consistently report that MALAT1 is up-regulated in human cancer tissues of various organ origins, particularly metastatic cancer tissues, and that high levels of MALAT1 in cancer tissues are associated with poor patient outcome. By interacting with EZH2, SUZ12, and a number of miRNAs, MALAT1 increases the expression of oncogenic factors, such as N-cadherin, β-catenin, c-Myc, ZEB1, and KRAS, decreases the expression of tumor suppressing factors, such as E-cadherin, p21, and p27, and induces cancer cell proliferation, migration and invasion *in vitro*, and tumor metastasis in mice. However, in these studies, the authors usually employ tumor samples from small cohorts of patients, and use MALAT1 antisense oligonucleotides, small interfering RNAs (siRNAs), or short hairpin RNA (shRNAs), without *in vitro* and *in vivo* rescue experiments, to demonstrate the specificity of the MALAT1 antisense oligonucleotides, siRNAs, and shRNAs. It is therefore difficult to make concrete conclusions.

By contrast, by analyzing seven independent human colorectal cancer tissue gene expression-patient prognosis datasets, RNA sequencing human breast cancer tissue gene expression-patient prognosis TCGA datasets and an Oncomine data-mining platform, Kwok *et al.* and Kim *et al.* have very recently found that MALAT1 gene expression is decreased in human colorectal and breast cancer tissues, and that a low level of MALAT1 is associated with decreased patient survival. By forming a RNA-protein complex with TEAD and down-regulating EpCAM, ITGB4 and VEGF expression, MALAT1 suppresses colorectal and breast cancer cell migration, invasion, and metastasis. Importantly, compared with control PyMT mouse mammary tumors, MALAT1-deficient PyMT mouse mammary tumors exhibit increased TEAD target gene expression and tumor metastasis, which can be reversed by genetic MALAT1 add-back.

It is therefore likely that MALAT1 suppresses colorectal and breast cancer cell migration, invasion, and metastasis. More comprehensive studies with multiple independent cohorts of human cancer tissues of various organ origins, *in vitro* and *in vivo* function and mechanism studies with rescue experiments are required to confirm the oncogenic or tumor suppressive role of MALAT1 in other cancers.

## Author Contributions

QC and CZ conceived and designed the review. QC, CZ and YJ wrote the manuscript. QC, CZ and YJ read and approved the final manuscript.

## Funding

This project was funded by the Natural Science Basic Research Program of Shaanxi Province (ID: 2017JM8180).

## Conflict of Interest

The authors declare that the research was conducted in the absence of any commercial or financial relationships that could be construed as a potential conflict of interest.
